# Peroxiredoxin 6 Regulates Glutathione Peroxidase 1-Medited Glutamine Synthase Preservation in the Hippocampus of Chronic Epilepsy Rats

**DOI:** 10.3390/antiox12010156

**Published:** 2023-01-09

**Authors:** Ji-Eun Kim, Hana Park, Tae-Cheon Kang

**Affiliations:** 1 Department of Anatomy and Neurobiolog, College of Medicine, Hallym University, Chuncheon 24252, Republic of Korea; 2 Institute of Epilepsy Research, Hallym University, Chuncheon 24252, Republic of Korea

**Keywords:** aiPLA2, autophagy, clasmatodendrosis, glutathione, GPx-1, GS, MJ33, NAC

## Abstract

Clasmatodendrosis (an autophagic astroglial degeneration) plays an important role in the regulation of spontaneous seizure duration but not seizure frequency or behavioral seizure severity in chronic epilepsy rats. Recently, it has been reported that N-acetylcysteine (NAC), a precursor to glutathione (GSH), attenuates clasmatodendritic degeneration and shortens spontaneous seizure duration in chronic epilepsy rats, although the underlying mechanisms of its anti-convulsive effects are not fully understood. To elucidate this, the present study was designed to investigate whether NAC affects astroglial glutamine synthase (GS) expression mediated by GSH peroxidase 1 (GPx1) and/or peroxiredoxin 6 (Prdx6) in the epileptic hippocampus. As compared to control animals, GS and GPx1 expressions were upregulated in reactive CA1 astrocytes of chronic epilepsy rats, while their expressions were significantly decreased in clasmatodendritic CA1 astrocytes and reactive astrocytes within the molecular layer of the dentate gyrus. Prdx6 expression was increased in reactive CA1 astrocytes as well as clasmatodendritic CA1 astrocytes. In the molecular layer of the dentate gyrus, Prdx6 expression levels were similar to those in control animals. NAC ameliorated clasmatodendrosis through the increment of GS and GPx1 expressions, while it abolished Prdx6 upregulation. 1-hexadecyl-3-(trifluoroethgl)-sn-glycerol-2 phosphomethanol (MJ33, a selective inhibitor of aiPLA2 activity of Prdx6) alleviated clasmatodendrosis by enhancing GPx1 and GS expressions in clasmatodendritic CA1 astrocytes without changing the Prdx6 level. NAC or MJ33 did not affect GS, GPx1 and Prdx6 expression in astrocytes within the molecular layer of the dentate gyrus. These findings indicate that upregulated aiPLA2 activity of Prdx6 may abolish GPx1-mediated GS preservation and lead to clasmatodendrosis in CA1 astrocytes, which would extend spontaneous seizure duration due to impaired glutamate-glutamine conversion regulated by GS. Therefore, the present data suggest that aiPLA2 activity of Prdx6 in astrocytes may be one of the upstream effectors of seizure duration in the epileptic hippocampus.

## 1. Introduction

Epilepsy is a chronic neurological disorder characterized by the recurrence of spontaneous seizures due to aberrant paroxysmal neuronal discharges. Temporal lobe epilepsy (TLE) is one of the most frequent and poorly responsive to antiepileptic drugs (AEDs), which results in devastating effects on patients, such as cognitive impairment and anxiety disorders [1,2]. Astrocyte plays a fundamental role in regulating neuronal activity and redox state in the brain [3]. Although astrocytes are invulnerable and activated in response to various insults, Alzheimer first reported irreversible astroglial degeneration characterized by extensive swollen cell bodies with vacuoles and disintegrated/beaded processes, and are termed as “clasmatodendrosis” by Cajal [4,5]. Clasmatodendrosis is caspase-independent (type II) programed astroglial death caused by excessive autophagic processing under various pathological conditions [6,7,8,9]. Although the underlying mechanisms of clasmatodendrosis are not fully understood, the impaired bioenergetics and severe intracellular acidosis lead to clasmatodendrosis [10,11,12].

Hydrogen peroxide (H_2_O_2_) is an inevitable by-product of mitochondrial respiration under physiological conditions. Since H_2_O_2_ is a source of reactive oxygen species (ROS), such as superoxide (O_2_^•−^) and hydroxyl radical (OH^•^), that lead to oxidative stress, the endogenous removal system of H_2_O_2_ is required for cell viability. Glutathione (GSH), a glycine-cysteine-glutamate tripeptide, is a major antioxidant that directly scavenges ROS in various cells. Cytosolic GSH peroxidase 1 (GPx1) and peroxiredoxin 6 (Prdx6) are GSH-dependent peroxidases, which are components of the enzymatic antioxidative defense system in the brain for maintaining the redox homeostasis of GSH [13,14]. GPx1 is highly expressed in neurons and astrocytes [15,16], while Prdx6 is a unique nonselenium-containing glutathione peroxidase (NSGPx), which acts as an acidic calcium-independent phospholipase (aiPLA2) as well as GPx in astrocytes but not in neurons [17,18].

Glutamine synthase (GS) is an astroglial enzyme that converts glutamate to glutamine, which regulates the glutamate-glutamine cycle between neurons and astrocytes. Thus, GS plays an important role in the maintenance of appropriate glutamate concentration in synapses, which prevents neuronal hyperexcitability and the subsequent excitotoxicity. Since glutamate is one of the rate-limiting precursors for GSH in astrocytes, GS also affects the GSH synthetic process [19,20,21]. Of interest, both GPx1 and Prdx6 prevent GS inactivation induced by oxidative stress [22,23]. Therefore, it is likely that GPx1 and Prdx6 may play an important role in the oxidative stress-induced neuronal excitability as well as redox homeostasis by maintaining GS activity to catalyze glutamate to glutamine.

Astrocytes show the regional-specific proportions of GS expression in the hippocampus of chronic epilepsy rats. Briefly, GS expression is strongly observed in reactive astrocytes within the stratum radiatum of the CA1 region (CA1 astrocytes). However, GS expression was significantly reduced in astrocytes within the molecular layer of the dentate gyrus. These different astroglial properties are related to the acquisition of the differential physiological properties of the epileptic hippocampus [24,25]. Recently, it has been reported that decreased GSH levels result in GPx1 downregulation in clasmatodendritic CA1 astrocytes and aberrant aiPLA2 activity of Prdx6 in reactive CA1 astrocytes, leading to clasmatodendrosis [26,27]. Furthermore, inhibition of clasmatodendritic degeneration by antioxidants, such as N-acetylcysteine (NAC, a glutathione precursor) and 2-cyano-3,12-dioxo-oleana-1,9(11)-dien-28-oic acid methyl ester (CDDO-Me), shortens spontaneous seizure duration in chronic epilepsy rats without affecting seizure frequency or behavioral seizure severity [27,28]. Therefore, it is likely that GPx1 and/or Prdx6-mediated GS regulation may regulate the duration of spontaneous seizures, which have been unreported.

Here, the present data demonstrate that GS and GPx1 expressions were upregulated in reactive CA1 astrocytes of chronic epilepsy rats, as compared to control animals, while their expressions were significantly decreased in clasmatodendritic CA1 astrocytes and reactive astrocytes within the molecular layer of the dentate gyrus. Prdx6 expression was increased in reactive CA1 astrocytes as well as clasmatodendritic CA1 astrocytes. In the molecular layer of the dentate gyrus, Prdx6 expression levels were similar to those in control animals. In the CA1 region, NAC ameliorated clasmatodendrosis through an increment of GS and GPx1 expressions while abolishing Prdx6 upregulation. 1-hexadecyl-3-(trifluoroethgl)-sn-glycerol-2 phosphomethanol (MJ33, a selective inhibitor of aiPLA2 activity of Prdx6) alleviated clasmatodendrosis by enhancing GPx1 and GS expressions in CA1 astrocytes without changing the Prdx6 level. In the molecular layer of the dentate gyrus, NAC or MJ33 did not affect GS, GPx1 and Prdx6 expression in astrocytes. To the best of our knowledge, these findings indicate for the first time that the distinct capability of aiPLA2 activity in Prdx6 may represent the differential properties of astrocytes in the epileptic hippocampus in a regional-specific manner. In addition, the present study provides novel evidence that enhancement of aiPLA2 activity of Prdx6 may abrogate GPx1-mediated GS preservation and lead to clasmatodendrosis in CA1 astrocytes, which would increase seizure duration by inhibiting glutamate-glutamine conversion.

## 2. Materials and Methods

### 2.1. Experimental Animals and Chemicals

Seven-week-old male Sprague-Dawley (SD) rats (200–250 g) housed in standard conditions (23–25 °C, 12 h light/dark cycle) with access to water and food ad libitum. The experiments were conducted in compliance with the Institutional Animal Care and Use Committee of Hallym University (Hallym 2021-30, approval date: 17 May 2021). All reagents were obtained from Sigma-Aldrich (St. Louis, MO, USA), except as noted.

### 2.2. Generation of Chronic Epilepsy Rats

Rats received LiCl (127 mg/kg, i.p.) 1 day before pilocarpine injection (30 mg/kg, i.p.). Twenty minutes before pilocarpine administration, atropine methylbromide (5 mg/kg i.p.) was treated to inhibit the peripheral effect of pilocarpine. Two hours after status epilepticus (SE) onset, diazepam (Valium; Hoffmann-la Roche, Neuilly-sur-Seine, France; 10 mg/kg, i.p.) was given to cease convulsion and repeated as needed. Control animals received saline. To detect spontaneous recurrent seizures indicating chronic epilepsy in rats, animals were video-monitored 8 h a day for 2 weeks after SE [11,26,27,28,29].

### 2.3. Drug Trials

Chronic epileptic rats were implanted with a brain infusion needle (Alzet, Cupertino, CA, USA) into the right lateral ventricle (coordinates: 1 mm posterior; 1.5 mm lateral; 3.5 mm depth) under isoflurane anesthesia (3% induction, 1.5–2% for surgery, and 1.5% maintenance in a 65:35 mixture of N_2_O:O_2_), and connected with an Alzet 1007D osmotic pump (Alzet, Cupertino, CA, USA) containing (1) vehicle and (2) MJ33 (50 μM). In some vehicle-infused animals, NAC (70 mg/kg) was administered once a day by intraperitoneal (i.p.) over 7 days [26,27]. The location of the needle was verified when the brain was sectioned.

### 2.4. Tissue Preparation and Immunohistochemistry

Seven days after surgery, rats were perfused with normal saline followed by 4% paraformaldehyde in 0.1 M phosphate buffer (PB, pH 7.4) via the ascending aorta under urethane anesthesia (1.5 g/kg, i.p.). The brains were cropped, postfixed with the same fixative overnight, and cryoprotected with 30% sucrose overnight. Thereafter, 30-μm-thick coronal sections were cut on a cryostat. Sections were incubated in blocking buffer (3% bovine serum albumin in PBS) for 30 min, and subsequently reacted with a mixed solution of primary antibodies (Table 1) overnight at room temperature. All antibodies were diluted with PBS containing 0.3% Triton X-After washing, sections were incubated with appropriate secondary antibodies conjugated with Brilliant Violet-421, Cy2- or Cy3-fluorescent dye (Jackson Immuno Research Laboratories, West Grove, PA, USA). A negative control test was performed with preimmune serum substituted for the primary antibody. The fluorescent intensity of each antibody was measured in the randomly selected 5 areas/animals (300 μm^2^/area) in the stratum radiatum of the CA1 region and the molecular layer of the dentate gyrus (5 sections from each animal, *n* = 7 in each group) with AxioVision Rel. 4.8 (Carl Zeiss Korea, Seoul, Republic of Korea) and ImageJ software. Fluorescent intensity was normalized by setting the mean background. For quantification of clasmatodendritic astrocytes (characterized by extensive swollen and vacuolized cell bodies), a cell count was performed in areas of interest (1 × 10^4^ μm^2^) of 10 sections per animal. GPx1, GS and Prdx6 fluorescent intensities were also measured in 2–3 reactive astrocytes or clasmatodendritic astrocytes randomly collected in each rat [26,27].

### 2.5. Data Analysis

To analyze the statistical significance of the data, Mann–Whitney test, Spearman test and Kruskal–Wallis test were applied. A *p*-value less than 0.05 was considered to be significant.

## 3. Results

### 3.1. GS and GPx1 Expressions Are Downregulated in Clasmatodendritic CA1 Astrocytes, But Not Reactive CA1 Astrocytes

**Figure 1:**
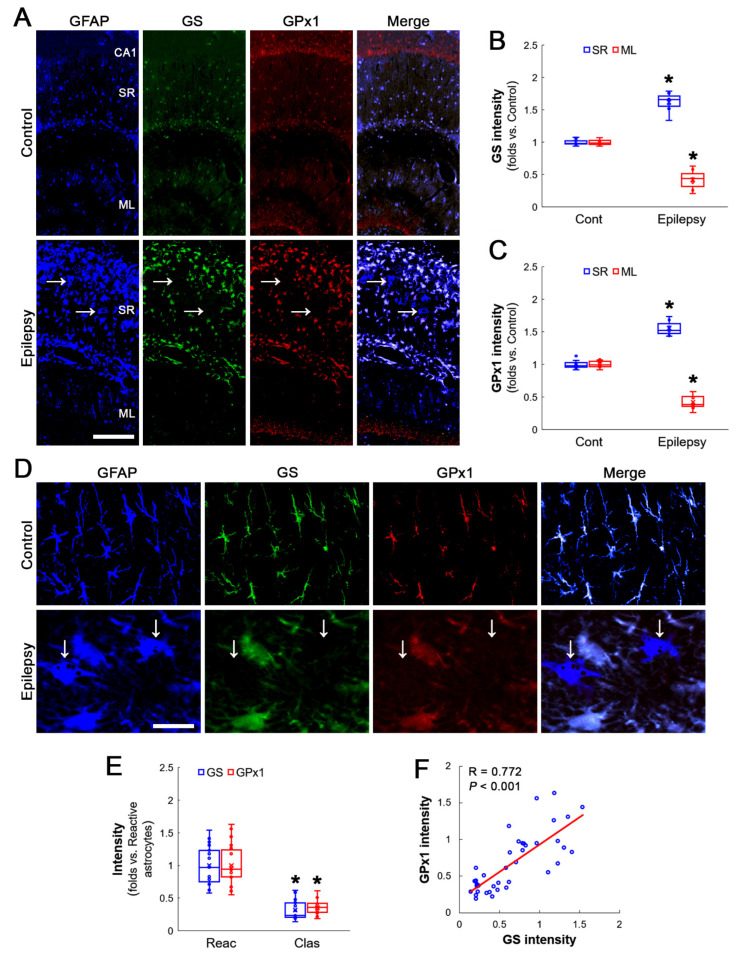
Alteration in GS and GPx1 expressions in the rat hippocampus of chronic epilepsy rats. GS expression is mainly observed in astrocytes (GFAP, an astroglial marker) under physiological conditions, while GPx1 is detected in CA1 pyramidal cells (CA1) as well as astrocytes. In chronic epilepsy rats, both GS and GPx1 expression were increased in the stratum radiatum (SR) of the CA1 region but diminished in the molecular layer (ML) of the dentate gyrus. These upregulations of GS and GPx1 expression were clearly detected in reactive CA1 astrocytes, but not in clasmatodendritic (vacuolized) CA1 astrocytes (arrows). (**A**) Representative photos of GFAP, GS and GPx1 expression in the hippocampus. Bar = 250 μm. (**B**,**C**) Quantification of GS (**B**) and GPx1 (**C**) intensity in the SR and the ML (* *p* < 0.05 vs. control animals; Mann–Whitney test; *n* = 7 rats, respectively). (**D**) Representative photos of triple fluorescent staining of GFAP, GS and GPx1 expression in CA1 astrocytes of chronic epilepsy rats. Bar = 12.5 μm. (**E**) Quantification of GS and GPx1 intensity in reactive and clasmatodendritic CA1 astrocytes of chronic epilepsy rats (* *p* < 0.05 vs. reactive astrocytes; Mann–Whitney test; *n* = 20 cells in 7 rats, respectively). (**F**) Linear regression analysis between GS and GPx1 expression in reactive and clasmatodendritic CA1 astrocytes of chronic epilepsy rats (*n* = 40 cells in 7 rats, Spearman test).

First, immunofluorescent studies using hippocampal tissues were performed to identify the cellular and regional alterations in GS and GPx1 expression. In chronic epilepsy rats, GS expression was upregulated to 1.52-fold of the control level in the stratum radiatum of the CA1 region of the hippocampus proper (Z = 3.134, *p* = 0.002, *n* = 7 rats, respectively, Mann–Whitney test; Figure 1A,B). Consistent with a previous study [26], GPx1 expression was also increased to 1.55-fold of the control level in this region (Z = 3.130, *p* = 0.002, *n* = 7 rats, respectively, Mann–Whitney test; Figure 1A,C). Although GS and GPx1 expressions were enhanced in reactive astrocytes, GS expression in clasmatodendritic astrocytes diminished 0.33-fold of the reactive astroglial level (Z = 5.347, *p* < 0.001, *n* = 20 cells in 7 rats, respectively, Mann–Whitney test), concomitant with GPx1 downregulation (Z = 5.371, *p* < 0.001, *n* = 20 cells in seven rats, respectively, Mann–Whitney test; Figure 1D,E). Thus, the astroglial GS expression showed a direct correlation with GPx1 expression (R = 0.772, *p* < 0.001, *n* = 40 cells in seven rats, Spearman test; Figure 1D,F).

Compatible with previous studies [24,25,27], reactive astrocytes in the molecular layer of the dentate gyrus of chronic epilepsy rats showed a very low GS expression level as compared to control animals (Z = 3.134, *p* = 0.002, *n* = 7 rats, respectively, Mann-Whitney test; Figure 1A,B). GPx1 expression level was also lower than that observed in control animals (Z = 3.137, *p* = 0.002, *n* = 7 rats, respectively, Mann–Whitney test; Figure 1A,C). These findings indicate that the decreased GS expression may be relevant to GPx1 downregulation in clasmatodendritic CA1 astrocytes.

### 3.2. Prdx6 Expression Is Increased in Both Reactive CA1 Astrocytes and Clasmatodendritic CA1 Astrocytes

**Figure 2:**
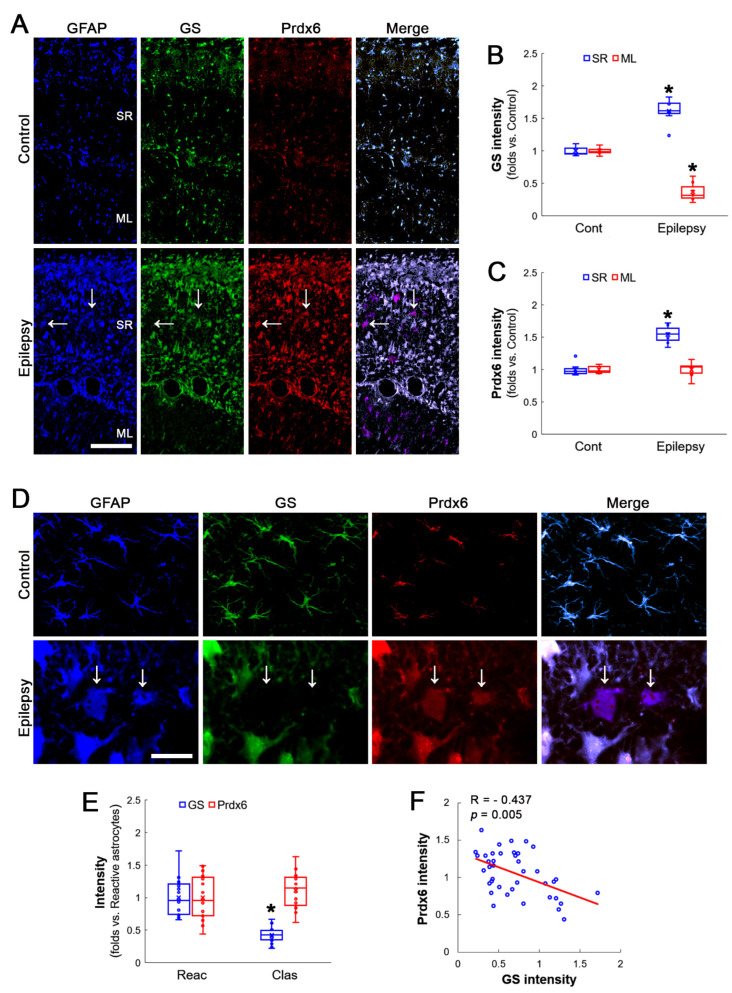
Alteration in GS and Prdx6 expressions in the rat hippocampus of chronic epilepsy rats. Both GS and Prdx6 expression were mainly detected in astrocytes (GFAP, an astroglial marker) under physiological conditions. In chronic epilepsy rats, Prdx6 expression is elevated in the stratum radiatum (SR) of the CA1 region but unaltered in the molecular layer (ML) of the dentate gyrus. Prdx6 upregulation is observed in reactive CA1 astrocytes as well as clasmatodendritic (vacuolized) CA1 astrocytes (arrows). (**A**) Representative photos of GFAP, GS and Prdx6 expression in the hippocampus. Bar = 250 μm. (**B**,**C**) Quantification of GS (**B**) and Prdx6 (**C**) intensity in the SR and the ML (* *p* < 0.05 vs. control animals; Mann–Whitney test; *n* = 7 rats, respectively). (**D**) Representative photos of triple fluorescent staining of GFAP, GS and Prdx6 expression in CA1 astrocytes of chronic epilepsy rats. Bar = 12.5 μm. (**E**) Quantification of GS and Prdx6 intensity in reactive and clasmatodendritic CA1 astrocytes of chronic epilepsy rats (* *p* < 0.05 vs. reactive astrocytes; Mann–Whitney test; *n* = 20 cells in 7 rats, respectively). (**F**) Linear regression analysis between GS and Prdx6 expression in reactive and clasmatodendritic CA1 astrocytes of chronic epilepsy rats (*n* = 40 cells in seven rats, Spearman test).

In a recent study [27], Prdx6 expression was increased in both reactive astrocytes as well as clasmatodendritic astrocytes. Consistent with this report, Prdx6 expression was increased in reactive astrocytes and clasmatodendritic astrocytes, showing GS downregulation within the stratum radiatum of the CA1 region of the hippocampus proper (Z = 3.130, *p* = 0.002, *n* = 7 rats, respectively, Mann–Whitney test; Figure 2A–C). Although GS expressions were diminished in clasmatodendritic astrocytes (Z = 5.385, *p* < 0.001, *n* = 20 cells in seven rats, respectively, Mann–Whitney test), there was no difference in Prdx6 expression between reactive astrocytes and clasmatodendritic astrocytes (Z = 1.042, *p* = 0.297, *n* = 20 cells in seven rats, respectively, Mann–Whitney test; Figure 2D,E). In contrast to GPx1 expression, GS expression was inversely proportional to Prdx6 expression (R = −0.437, *p* = 0.005, *n* = 40 cells in seven rats, Spearman test; Figure 2D,F). These findings indicate that unlike in vitro model using *Escherichia coli* [22] Prdx6 upregulation may not prevent GS inactivation but suppress GS expression.

In reactive astrocytes in the molecular layer of the dentate gyrus of chronic epilepsy rats, Prdx6 expression level was similar to that in control animals (Z = 0.128, *p* = 0.898, *n* = 7 rats, respectively, Mann–Whitney test), while GS expression was lower than that of control animals (Z = 3.130, *p* = 0.002, *n* = 7 rats, respectively, Mann–Whitney test; Figure 2A–C).

### 3.3. NAC Enhances GS and GPx1 Expressions, But Reduces Prdx6 Level in Clasmatodendritic CA1 Astrocytes

GSH deprivation leads to GPx1 downregulation and deteriorates clasmatodendritic astrocytes. In addition, NAC protects astrocytes from clasmatodendritic degeneration accompanied by GPx1 upregulation [26]. Since GPx1 plays a protective role in oxidative stress-induced GS inactivation [23], the effect of NAC on GS expression in CA1 astrocytes was explored.

**Figure 3:**
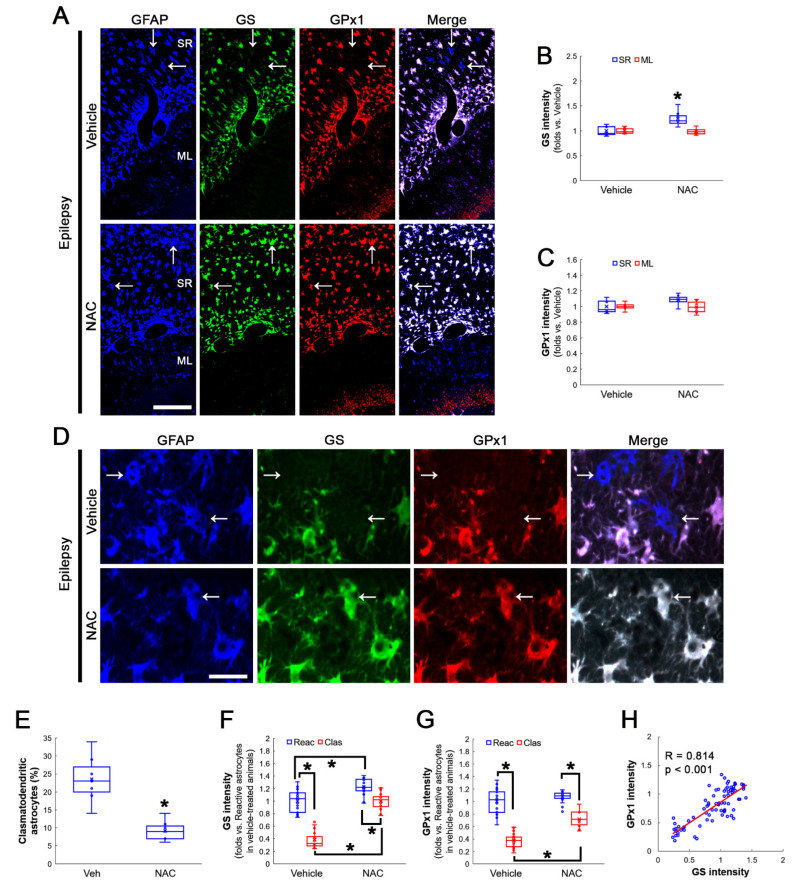
Effects of NAC on GS and GPx1 expressions and clasmatodendritic degeneration of CA1 astrocytes in chronic epilepsy rats. As compared to a vehicle, NAC upregulates GS expression in reactive astrocytes and clasmatodendritic astrocytes within the stratum radiatum (SR) of the CA1 region, while it enhances GPx1 expression only in clasmatodendritic (vacuolized) CA1 astrocytes (arrows). NAC also attenuates clasmatodendrosis in CA1 astrocytes. However, NAC does not affect GS and GPx1 expressions in the molecular layer (ML) of the dentate gyrus. (**A**) Representative photos of GFAP, GS and GPx1 expression in the hippocampus. Bar = 250 μm. (**B**,**C**) Quantification of GS (**B**) and GPx1 (**C**) intensity in the SR and the ML following NAC treatment (* *p* < 0.05 vs. control animals; Mann–Whitney test; *n* = 7 rats, respectively). (**D**) Representative photos of triple fluorescent staining of GFAP, GS and Prdx6 expression in CA1 astrocytes of chronic epilepsy rats. Bar = 12.5 μm. (**E**) Quantification of clasmatodendritic degeneration in CA1 astrocytes (* *p* < 0.05 vs. vehicle; Mann–Whitney test; *n* = 7 rats, respectively). (**F**,**G**) Quantification of GS (**F**) and GPx1 (**G**) intensity in reactive and clasmatodendritic CA1 astrocytes of chronic epilepsy rats following NAC treatment (* *p* < 0.05 vs. reactive astrocytes; Mann–Whitney test; *n* = 20 cells in seven rats, respectively). (**H**) Linear regression analysis between GS and GPx1 expression in reactive and clasmatodendritic CA1 astrocytes of chronic epilepsy rats following NAC treatment (*n* = 80 cells in 14 rats, Spearman test).

NAC increased GS expression levels to 1.25-fold of vehicle-treated animal levels in the stratum radiatum of the CA1 region of the hippocampus proper (Z = 2.689, *p* = 0.007, *n* = 7 rats, respectively, Mann–Whitney test; Figure 3A,B). NAC slightly enhanced, but not significantly, GPx1 expression in this region (Z = 1.853, *p* = 0.064, *n* = 7 rats, respectively, Mann–Whitney test; Figure 3A,C). NAC did not influence GS and GPx1 expression in the molecular layer of the dentate gyrus (Figure 3A–C). In vehicle-treated animals, the fraction of clasmatodendritic astrocytes in total astrocytes was 23.6%. NAC decreased the fraction of clasmatodendritic CA1 astrocytes to 10% (Z = 3.073, *p* = 0.002, *n* = 7 rats, respectively, Mann–Whitney test; Figure 3D,E). NAC enhanced GS expression in reactive astrocytes and clasmatodendritic (vacuolized) astrocytes (*χ^2^*_(3)_ = 57.225, *p* < 0.001, *n* = 20 cells in seven rats, respectively, Kruskal–Wallis test; Figure 3D,F). NAC also increased GPx1 expression in clasmatodendritic astrocytes (*χ^2^*_(3)_ = 57.948, *p* < 0.001, *n* = 20 cells in seven rats, respectively, Kruskal–Wallis test), but not in reactive astrocytes (*p* = 0.178, Tukey *post-hoc* test; Figure 3D,G). Thus, GS and GPx1 expressions showed a direct proportion (R = 0.814, *p* < 0.001, *n* = 80 cells in 14 rats, Spearman test; Figure 3D,H).

**Figure 4:**
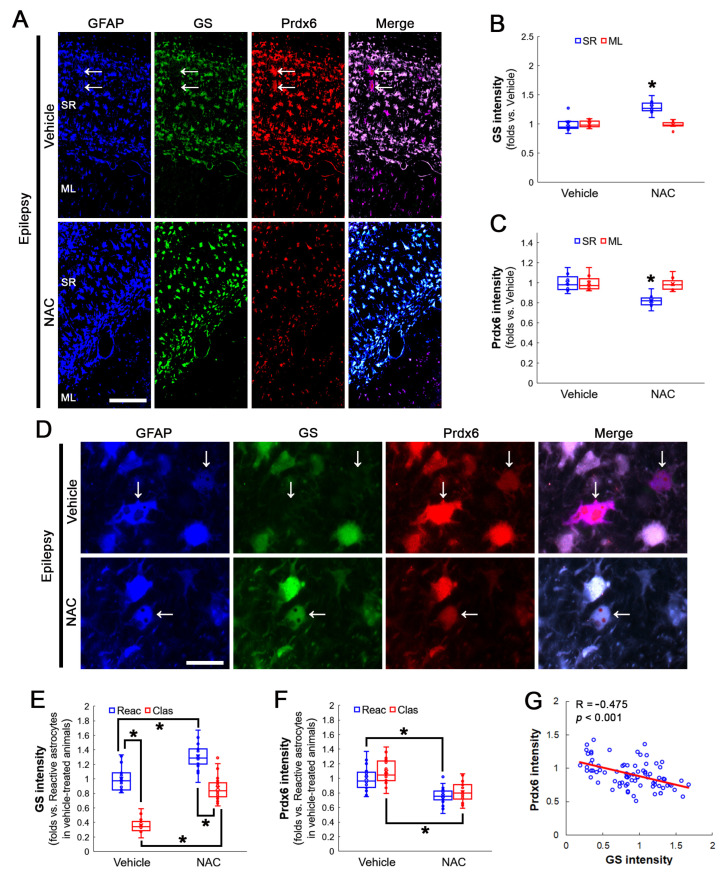
Effects of NAC on GS and Prdx6 expressions and clasmatodendritic degeneration of CA1 astrocytes in chronic epilepsy rats. As compared to a vehicle, NAC increases GS levels in clasmatodendritic (vacuolized) astrocytes (arrows) within the stratum radiatum (SR) of the CA1 region, while decreasing astroglial Prdx6 levels in this region. NAC does not affect GS and Prdx6 expressions in the molecular layer (ML) of the dentate gyrus. (**A**) Representative photos of GFAP, GS and Prdx6 expression in the hippocampus. Bar = 250 μm. (**B**,**C**) Quantification of GS (**B**) and Prdx6 (**C**) intensity in the SR and the ML following NAC treatment (* *p* < 0.05 vs. control animals; Mann–Whitney test; *n* = 7 rats, respectively). (**D**) Representative photos of triple fluorescent staining of GFAP, GS and Prdx6 expression in CA1 astrocytes of chronic epilepsy rats. Bar = 12.5 μm. (**E**,**F**) Quantification of GS (**E**) and Prdx6 (**F**) intensity in reactive and clasmatodendritic CA1 astrocytes of chronic epilepsy rats following NAC treatment (* *p* < 0.05 vs. reactive astrocytes; Mann–Whitney test; *n* = 20 cells in seven rats, respectively). (**G**) Linear regression analysis between GS and Prdx6 expression in reactive and clasmatodendritic CA1 astrocytes of chronic epilepsy rats following NAC treatment (*n* = 80 cells in 14 rats, Spearman test).

Consistent with a previous study [27], NAC reduced Prdx6 level to 0.82-fold of vehicle-treated animal levels in the stratum radiatum of the CA1 region of the hippocampus proper (Z = 2.814, *p* = 0.005, *n* = 7 rats, respectively, Mann–Whitney test), concomitant with enhanced GS expression (Z = 2.689, *p* = 0.007, *n* = 7 rats, respectively, Mann–Whitney test; Figure 4A–C). NAC did not affect Prdx6 levels in the molecular layer of the dentate gyrus (Figure 4A–C). Although NAC increased GS expression in reactive astrocytes and clasmatodendritic astrocytes (*χ^2^*_(3)_ = 60.697, *p* < 0.001, *n* = 20 cells in seven rats, respectively, Kruskal–Wallis test; Figure 4D,E), it diminished Prdx6 expression in clasmatodendritic astrocytes as well as reactive astrocytes (*χ^2^*_(3)_ = 38.954, *p* < 0.001, *n* = 20 cells in seven rats, respectively, Kruskal–Wallis test; Figure 4D,F). Therefore, GS and Prdx6 expressions showed an inverse proportion (R = −0.475, *p* < 0.001, *n* = 80 cells in 14 rats, Spearman test; Figure 4D,G).

### 3.4. Inhibition of aiPLA2 Activity of Prdx6 Increases GS and GPx1 Expressions in Clasmatodendritic CA1 Astrocytes without Affecting Prdx6 Levels

Prdx6 has aiPLA2 activity as well as GPx properties [18]. This aiPLA2 activity of Prdx6 is involved in cell damage [30,31]. Indeed, increased aiPLA2 activity of Prdx6 leads to clasmatodendrosis in CA1 astrocytes [27]. Thus, it is likely that the aiPLA2 activity of Prdx6 would affect the downregulation of GS and GPx1 expression in clasmatodendritic CA1 astrocytes. To confirm this, MJ33 (a selective inhibitor of aiPLA2 activity of Prdx6) was applied to chronic epilepsy rats.

**Figure 5:**
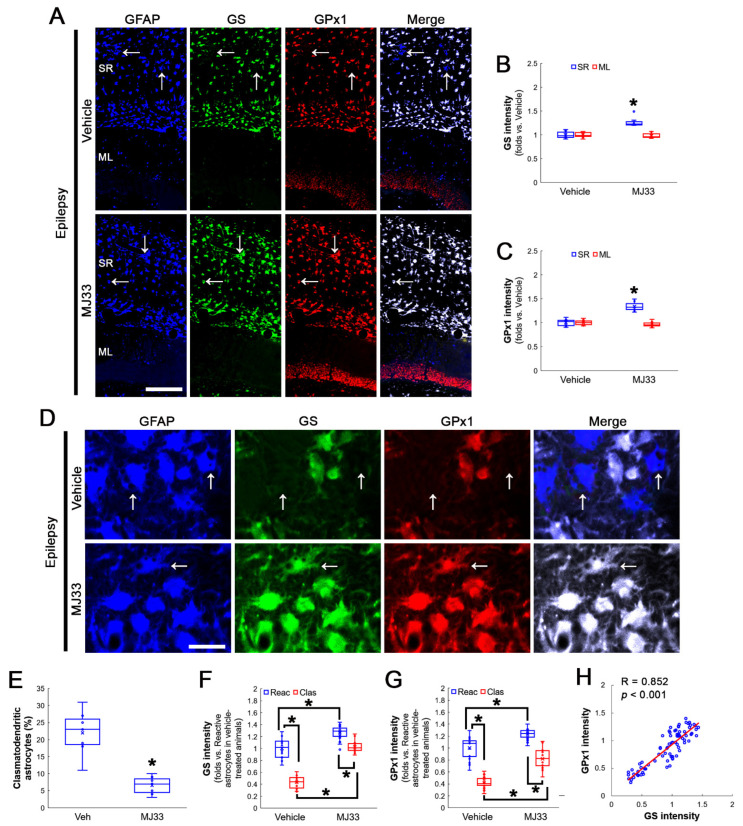
Effects of MJ33 on GS and GPx1 expressions and clasmatodendritic degeneration of CA1 astrocytes in chronic epilepsy rats. As compared to a vehicle, MJ33 upregulates both GS and GPx1 expression in reactive astrocytes and clasmatodendritic (vacuolized) astrocytes (arrows) within the stratum radiatum (SR) of the CA1 region. NAC also ameliorates clasmatodendritic degeneration. MJ33 does not change GS and GPx1 expressions in the molecular layer (ML) of the dentate gyrus. (**A**) Representative photos of GFAP, GS and GPx1 expression in the hippocampus. Bar = 250 μm. (**B**,**C**) Quantification of GS (**B**) and GPx1 (**C**) intensity in the SR and the ML following MJ33 treatment (* *p* < 0.05 vs. control animals; Mann–Whitney test; *n* = 7 rats, respectively). (**D**) Representative photos of triple fluorescent staining of GFAP, GS and Prdx6 expression in CA1 astrocytes of chronic epilepsy rats. Bar = 12.5 μm. (**E**) Quantification of clasmatodendritic degeneration in CA1 astrocytes (* *p* < 0.05 vs. vehicle; Mann–Whitney test; *n* = 7 rats, respectively). (**F**,**G**) Quantification of GS (**F**) and GPx1 (**G**) intensity in reactive and clasmatodendritic CA1 astrocytes of chronic epilepsy rats following MJ33 treatment (* *p* < 0.05 vs. reactive astrocytes; Mann–Whitney test; *n* = 20 cells in seven rats, respectively). (**H**) Linear regression analysis between GS and GPx1 expression in reactive and clasmatodendritic CA1 astrocytes of chronic epilepsy rats following MJ33 treatment (*n* = 80 cells in 14 rats, Spearman test).

MJ33 increased GS expression levels to 1.26-fold of vehicle-treated animal levels in the stratum radiatum of the CA1 region of the hippocampus proper (Z = 3.07, *p* = 0.002, *n* = 7 rats, respectively, Mann–Whitney test; Figure 5A,B), concomitant with GPx1 upregulation (Z = 3.13, *p* = 0.002, *n* = 7 rats, respectively, Mann–Whitney test; Figure 5A,C). MJ33 did not influence GS and GPx1 expression in the molecular layer of the dentate gyrus (Figure 5A–C). In vehicle-treated animals, the fraction of clasmatodendritic astrocytes in total astrocytes was 22%. NAC decreased the fraction of clasmatodendritic CA1 astrocytes to 6.57% (Z = 3.13, *p* = 0.002, *n* = 7 rats, respectively, Mann–Whitney test; Figure 5D,E). MJ33 increased GS (*χ^2^*_(3)_ = 59.709, *p* < 0.001, *n* = 20 cells in seven rats, respectively, Kruskal–Wallis test; Figure 5D,F) and GPx1 expressions (*χ^2^*_(3)_ = 64.185, *p* < 0.001, *n* = 20 cells in seven rats, respectively, Kruskal–Wallis test; Figure 5D,G) in clasmatodendritic astrocytes as well as reactive astrocytes. Thus, GS and GPx1 expressions showed direct proportion (R = 0.852, *p* < 0.001, *n* = 80 cells in 14 rats, Spearman test; Figure 5D,H).

**Figure 6:**
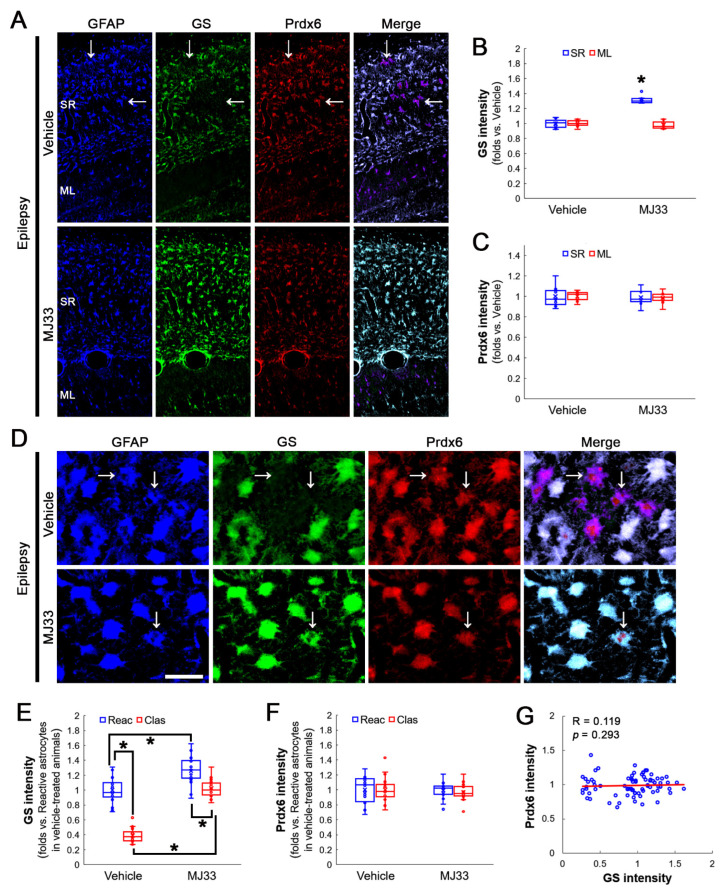
Effects of MJ33 on GS and Prdx6 expressions and clasmatodendritic degeneration of CA1 astrocytes in chronic epilepsy rats. As compared to a vehicle, MJ33 increases GS levels in reactive astrocytes and clasmatodendritic (vacuolized) astrocytes (arrows) within the stratum radiatum (SR) of the CA1 region, while it does not influence astroglial Prdx6 levels in this region. MJ33 does not influence GS and Prdx6 expressions in the molecular layer (ML) of the dentate gyrus. (**A**) Representative photos of GFAP, GS and Prdx6 expression in the hippocampus. Bar = 250 μm. (**B**,**C**) Quantification of GS (**B**) and Prdx6 (**C**) intensity in the SR and the ML following MJ33 treatment (* *p* < 0.05 vs. control animals; Mann–Whitney test; *n* = 7 rats, respectively). (**D**) Representative photos of triple fluorescent staining of GFAP, GS and Prdx6 expression in CA1 astrocytes of chronic epilepsy rats. Bar = 12.5 μm. (**E**,**F**) Quantification of GS (**E**) and Prdx6 (**F**) intensity in reactive and clasmatodendritic CA1 astrocytes of chronic epilepsy rats following MJ33 treatment (* *p* < 0.05 vs. reactive astrocytes; Mann–Whitney test; *n* = 20 cells in seven rats, respectively). (**G**) Linear regression analysis between GS and Prdx6 expression in reactive and clasmatodendritic CA1 astrocytes of chronic epilepsy rats following MJ33 treatment (*n* = 80 cells in 14 rats, Spearman test).

Consistent with previous studies [27,32], MJ33 did not affect Prdx6 levels in the stratum radiatum of the CA1 region of the hippocampus proper, while it enhanced GS expression (Z = 3.07, *p* = 0.002, *n* = 7 rats, respectively, Mann–Whitney test; Figure 6A–C). MJ33 did not influence Prdx6 expression in the molecular layer of the dentate gyrus (Figure 6A–C). MJ33 enhanced GS expression in reactive- and clasmatodendritic CA1 astrocytes (*χ^2^*_(3)_ = 55.082, *p* < 0.001, *n* = 20 cells in seven rats, respectively, Kruskal–Wallis test; Figure 6D,E) without altering Prdx6 expression (*χ^2^*_(3)_ = 1.233, *p* = 0.745, *n* = 20 cells in seven rats, respectively, Kruskal–Wallis test; Figure 6D,F). Linear regression analysis of GS and Prdx6 expression showed no correlation (R = 0.119, *p* = 0.293, *n* = 80 cells in 14 rats, Spearman test; Figure 6D,G).

## 4. Discussion

The novel findings in the present study are that the ROS-Prdx6-GPx1-GS axis in CA1 astrocytes might be relevant to clasmatodendritic degeneration (autophagic astroglial degeneration) and the increased seizure duration in chronic epilepsy rats by inhibiting glutamate-glutamine conversion.

Although oxidative stress plays an important role in spontaneous seizure activity and acquired epilepsy [27,28,33,34], the underlying mechanisms of antioxidant-induced regulation of seizure activity are not fully understood. Oxidative stress induces clasmatodendrosis that is lysosome-derived autophagic astroglial death. Indeed, vacuoles in clasmatodendritic astrocytes are identified as lysosomal-associated membrane protein 1 (LAPM-1). Ultrastructural studies also confirmed that clasmatodendritic astrocytes showed autophagocytosis and ubiquitin proteasome system (UPS)-mediated degeneration [6,7,8,9,11,12,26,27,28,35]. Recently, it has been reported that oxidative stress induces specificity protein 1 (Sp1)-mediated Prdx6 upregulation, which increases its aiPLA2 activity that dominates over GPx activity in CA1 astrocytes, and results in clasmatodendrosis [27]. In addition, NAC-induced Prdx6 downregulation and MJ33-induced inhibition of its aiPLA2 activity shorten seizure duration in chronic epilepsy rats [27]. Prdx6 is a GSH-dependent peroxidase and the major enzyme for reduction in oxidized phospholipids. The aiPLA2 activity of Prdx6 is also relevant for the repair of peroxidized cell membranes at physiological pH (pH 7), which is increased by GSH [14,36]. Since Prdx6 abolishes an elevated intracellular Ca^2+^ concentration, which suppresses necrosis and apoptosis in astrocytes [36], Prdx6 upregulation is one of the defense mechanisms in astrocytes against oxidative damage [18,37,38,39]. However, Prdx6 is also involved in the proinflammatory signals after stroke [40,41,42,43]. Furthermore, intracellular acidosis leads to clasmatodendrosis and compels Prdx6 to act as aiPLA2 rather than GPx in the cytoplasm, which elicits NADPH oxidase-mediated ROS formation [10,32,44]. Compatible with a previous study [27], the present study reveals that NAC attenuates clasmatodendritic degeneration by reducing Prdx6 expression. Furthermore, inhibition of aiPLA2 activity of Prdx6 by MJ33 mitigated this autophagic astroglial death without altering Prdx6 expression. Considering that Prdx6 supports optimal NADPH oxidase activity and the increased aiPLA2 activity of Prdx6 leads to NADPH oxidase-mediated oxidative stress [30,31,45,46], these findings indicate that oxidative stress-induced Prdx6 activation as aiPLA2 may lead to further oxidative stress in reactive astrocytes, which would elicit clasmatodendritic degeneration. Furthermore, the present data also demonstrate that both GS and GPx1 expressions restored in vacuolized astrocytes by NAC or MJ33 treatment indicate that GS and GPx1 may prevent or delay clasmatodendritic degeneration from oxidative stress. Therefore, it is plausible that Prdx6-mediated NADPH oxidase would result in clasmatodendrosis by generating excessive ROS and subsequently diminishing GPx1-mediated GS preservation. Adversely, GS or GPx1 downregulation would exert the increased Prdx6 expression in clasmatodendritic astrocytes. However, Prdx6 is unaffected by GPx1 deficiency [47], while the Prdx family gene expressions are relevant to GPx1 expression [48]. Therefore, the present data suggest that increased aiPLA2 activity of Prdx6 in the clasmatodendritic process may elicit downregulation of GS and GPx1 expression.

Reactive astrocytes in distinct parts of the hippocampoentorhinal complex in chronic epilepsy rats show different characteristics of GS expression. Reactive astrocytes in the stratum radiatum of the CA1 region show GS upregulation, while in the dentate gyrus, astrocytes contain very low GS expression. This different GS expression is an aspect of the distinct astroglial capability for ictogenesis in the epileptic hippocampoentorhinal complex [25]. In the present study, GS expression was upregulated in the stratum radiatum of the CA1 region of the hippocampus proper in chronic epilepsy rats, while its expression was markedly diminished in clasmatodendritic CA1 astrocytes. GS is required for the conversion of glutamate and ammonia to glutamine in astrocytes. Thus, GS inhibition causes the accumulation of glutamate and ammonia in astrocytes [49]. Since ammonia contributes to ROS formation in astrocytes [50,51,52], it is presumed that downregulated GS expression in clasmatodendritic astrocytes would facilitate excessive autophagic processes due to ammonia-mediated ROS generation in astrocytes. In contrast, ammonia inhibits autophagy by increasing the intracellular and lysosomal pH, independent of a GS-mediated process [52]. Thus, it is also considerable that GS downregulation might be an adaptive response to the inhibition of aiPLA2 activity of Prdx6 in clasmatodendritic astrocytes via increased intracellular pH. If true, clasmatodendritic astrocytes would show strong GS expression. However, the present data reveal that GS expression in clasmatodendrosis was lower than that in reactive astrocytes. Therefore, it is likely that GS downregulation in clasmatodendritic astrocytes may not be a cause, but a consequence of clasmatodendritic degeneration induced by the aberrant activation of aiPLA2 properties of Prdx6.

Concomitant with diminished GS expression, the present data show GPx1 downregulation in clasmatodendritic CA1 astrocytes. Furthermore, NAC enhanced both GS and GPx1 expressions in clasmatodendritic astrocytes in the stratum radiatum of the CA1 region of the hippocampus proper and attenuated clasmatodendritic degeneration. The specific activity of GS is strongly diminished by H_2_O_2_ in astrocytes, which is protected by GPx1 [23]. Therefore, decreased GS levels may be attributed to GPx1 downregulation in clasmatodendritic astrocytes, which would increase glutamate concentration due to impaired glutamate-glutamine conversion and, in turn, extend seizure duration.

In the present study, reactive astrocytes in the molecular layer of the dentate gyrus showed very low GS and GPx1 expressions, while Prdx6 expression levels were similar to those in control animals. Unlike the CA1 region, SE leads to massive astroglial apoptosis in the molecular layer of the dentate gyrus [24,25,53,54]. This regional specific astroglial apoptosis is independent of hemodynamics, indicating the distinct properties of astrocytes in regional specific manners [53,54]. After massive astroglial loss, reactive astrocytes in the molecular layer of the dentate gyrus are derived from newly generated astrocytes in the subgranular zone, which show immature properties. Therefore, the present data indicate that the altered GS, GPx1 and Prdx6 expressions in the molecular layer of the dentate gyrus may be a consequence of astroglial apoptosis followed by gliogenesis rather than the dedifferentiation of naïve astrocytes. In this region, furthermore, the expression levels of GS, GPx1 and Prdx6 are unaffected by NAC and MJ33. These findings also represent the acquisition of the differential properties of immature, newly generated astrocytes.

## 5. Conclusions

The present study reveals for the first time that aiPLA2 activity of Prdx6 diminished GS and GPx1 expressions in response to oxidative stress, which exerted autophagic astroglial degeneration in the epileptic hippocampus. In addition, restoration of GS and GPx1 expression by NAC or MJ33 attenuated or delayed clasmatodendritic degeneration. Therefore, the present data suggest that inhibition of aiPLA2 activity by Prdx6 may be a strategy to increase astroglial viability against oxidative stress, which would shorten the duration of spontaneous seizures.

## Figures and Tables

**Table 1:** Primary antibodies used in the present study.

Antigen	Host	Manufacturer (Catalog Number)	Dilution Used
Prdx6	Sheep	Biosensis (S-073-100)	1:1000
GS	Mouse	Millipore (#MAB302)	1:500
GPx1	Sheep	Biosensis (S-072-100)	1:2000
GFAP	Rabbit	Millipore (#AB5804)	1:500

## Data Availability

The data are contained within the article.

## References

[1] Vinti V., Dell’Isola G.B., Tascini G., Mencaroni E., Cara G.D., Striano P., Verrotti A. (2021). Temporal Lobe Epilepsy and Psychiatric Comorbidity. Front. Neurol..

[2] You J., Huang H., Chan C.T.Y., Li L. (2022). Pathological Targets for Treating Temporal Lobe Epilepsy: Discoveries From Microscale to Macroscale. Front. Neurol..

[3] Chen Y., Swanson R.A. (2003). Astrocytes and brain injury. J. Cereb. Blood Flow Metab..

[4] Penfield W., Cowdry E.V. (1928). Neuroglia and microglia—The interstitial tissue of the central nervous system. Special Cytology, the Form and Function of the Cell in Health and Disease.

[5] Duchen L.W., Adams J.H., Duchen L.W. (1992). General pathology of neurons and neuroglia. Greenfield’s Neuropathology.

[6] Ryu H.J., Kim J.E., Yeo S.I., Kim D.W., Kwon O.S., Choi S.Y., Kang T.C. (2011). F-actin depolymerization accelerates clasmatodendrosis via activation of lysosome-derived autophagic astroglial death. Brain Res. Bull..

[7] Thorburn A. (2008). Apoptosis and autophagy: Regulatory connections between two supposedly different processes. Apoptosis.

[8] Sakai K., Fukuda T., Iwadate K. (2013). Beading of the astrocytic processes (clasmatodendrosis) following head trauma is associated with protein degradation pathways. Brain Inj..

[9] Bouchat J., Gilloteaux J., Suain V., Van Vlaender D., Brion J.P., Nicaise C. (2019). Ultrastructural Analysis of Thalamus Damages in a Mouse Model of Osmotic-Induced Demyelination. Neurotox. Res..

[10] Hulse R.E., Winterfield J., Kunkler P.E., Kraig R.P. (2001). Astrocytic clasmatodendrosis in hippocampal organ culture. Glia.

[11] Kim J.E., Hyun H.W., Min S.J., Kang T.C. (2017). Sustained HSP25 Expression Induces Clasmatodendrosis via ER Stress in the Rat Hippocampus. Front. Cell Neurosci..

[12] Kim J.E., Ko A.R., Hyun H.W., Min S.J., Kang T.C. (2018). P2RX7-MAPK1/2-SP1 axis inhibits MTOR independent HSPB1-mediated astroglial autophagy. Cell Death Dis..

[13] Brigelius-Flohé R. (1999). Tissue-specific functions of individual glutathione peroxidases. Free Radic. Biol. Med..

[14] Liu G., Feinstein S.I., Wang Y., Dodia C., Fisher D., Yu K., Ho Y.S., Fisher A.B. (2010). Comparison of glutathione peroxidase 1 and peroxiredoxin 6 in protection against oxidative stress in the mouse lung. Free Radic. Biol. Med..

[15] Trépanier G., Furling D., Puymirat J., Mirault M.E. (1996). Immunocytochemical localization of seleno-glutathione peroxidase in the adult mouse brain. Neuroscience.

[16] Power J.H., Blumbergs P.C. (2009). Cellular glutathione peroxidase in human brain: Cellular distribution, and its potential role in the degradation of Lewy bodies in Parkinson’s disease and dementia with Lewy bodies. Acta Neuropathol..

[17] Goemaere J., Knoops B. (2012). Peroxiredoxin distribution in the mouse brain with emphasis on neuronal populations affected in neurodegenerative disorders. J. Comp. Neurol..

[18] Liao J., Zhang Y., Chen X., Zhang J. (2021). The Roles of Peroxiredoxin 6 in Brain Diseases. Mol. Neurobiol..

[19] Dringen R. (2000). Metabolism and functions of glutathione in brain. Prog. Neurobiol..

[20] Dringen R., Brandmann M., Hohnholt M.C., Blumrich E.M. (2015). Glutathione-dependent detoxification processes in astrocytes. Neurochem. Res..

[21] Anderson C.M., Swanson R.A. (2000). Astrocyte glutamate transport: Review of properties, regulation, and physiological functions. Glia.

[22] Merkulova M.I., Shuvaeva T.M., Radchenko V.V., Yanin B.A., Bondar A.A., Sofin A.D., Lipkin V.M. (2002). Recombinant human peroxiredoxin VI: Preparation and protective properties in vitro. Biochemistry.

[23] Knorpp T., Robinson S.R., Crack P.J., Dringen R. (2006). Glutathione peroxidase-1 contributes to the protection of glutamine synthetase in astrocytes during oxidative stress. J. Neural. Transm..

[24] Kang T.C., Kim D.S., Kwak S.E., Kim J.E., Won M.H., Kim D.W., Choi S.Y., Kwon O.S. (2006). Epileptogenic roles of astroglial death and regeneration in the dentate gyrus of experimental temporal lobe epilepsy. Glia.

[25] Kim D.S., Kim J.E., Kwak S.E., Choi K.C., Kim D.W., Kwon O.S., Choi S.Y., Kang T.C. (2008). Spatiotemporal characteristics of astroglial death in the rat hippocampo-entorhinal complex following pilocarpine-induced status epilepticus. J. Comp. Neurol..

[26] Kim J.E., Lee D.S., Kim T.H., Kang T.C. (2022). Glutathione Regulates GPx1 Expression during CA1 Neuronal Death and Clasmatodendrosis in the Rat Hippocampus following Status Epilepticus. Antioxidants.

[27] Kim J.E., Lee D.S., Kang T.C. (2022). Sp1-Mediated Prdx6 Upregulation Leads to Clasmatodendrosis by Increasing Its aiPLA2 Activity in the CA1 Astrocytes in Chronic Epilepsy Rats. Antioxidants.

[28] Kim J.E., Kang T.C. (2021). CDDO-Me Attenuates Astroglial Autophagy via Nrf2-, ERK1/2-SP1- and Src-CK2-PTEN-PI3K/AKT-Mediated Signaling Pathways in the Hippocampus of Chronic Epilepsy Rats. Antioxidants.

[29] Kim J.E., Lee D.S., Park H., Kim T.H., Kang T.C. (2021). Inhibition of AKT/GSK3β/CREB Pathway Improves the Responsiveness to AMPA Receptor Antagonists by Regulating GRIA1 Surface Expression in Chronic Epilepsy Rats. Biomedicines.

[30] Ellison M.A., Thurman G.W., Ambruso D.R. (2012). Phox activity of differentiated PLB-985 cells is enhanced, in an agonist specific manner, by the PLA2 activity of Prdx6-PLA. Eur. J. Immunol..

[31] Krishnaiah S.Y., Dodia C., Feinstein S.I., Fisher A.B. (2013). p67(phox) terminates the phospholipase A(2)-derived signal for activation of NADPH oxidase (NOX2). FASEB J..

[32] Kwon J., Wang A., Burke D.J., Boudreau H.E., Lekstrom K.J., Korzeniowska A., Sugamata R., Kim Y.S., Yi L., Ersoy I. (2016). Peroxiredoxin 6 (Prdx6) supports NADPH oxidase 1 (Nox1)-based superoxide generation and cell migration. Free Radic. Biol. Med..

[33] Pauletti A., Terrone G., Shekh-Ahmad T., Salamone A., Ravizza T., Rizzi M., Pastore A., Pascente R., Liang L.P., Villa B.R. (2019). Targeting oxidative stress improves disease outcomes in a rat model of acquired epilepsy. Brain.

[34] González-Reyes S., Santillán-Cigales J.J., Jiménez-Osorio A.S., Pedraza-Chaverri J., Guevara-Guzmán R. (2016). Glycyrrhizin ameliorates oxidative stress and inflammation in hippocampus and olfactory bulb in lithium/pilocarpine-induced status epilepticus in rats. Epilepsy Res..

[35] Ryu H.J., Kim J.E., Yeo S.I., Kang T.C. (2011). p65/RelA-Ser529 NF-κB subunit phosphorylation induces autophagic astroglial death (Clasmatodendrosis) following status epilepticus. Cell Mol. Neurobiol..

[36] Zhou S., Dodia C., Feinstein S.I., Harper S., Forman H.J., Speicher D.W., Fisher A.B. (2018). Oxidation of Peroxiredoxin 6 in the Presence of GSH Increases its Phospholipase A₂ Activity at Cytoplasmic pH. Antioxidants.

[37] Turovsky E.A., Varlamova E.G., Plotnikov E.Y. (2021). Mechanisms Underlying the Protective Effect of the Peroxiredoxin-6 Are Mediated via the Protection of Astrocytes during Ischemia/Reoxygenation. Int. J. Mol. Sci..

[38] Krapfenbauer K., Engidawork E., Cairns N., Fountoulakis M., Lubec G. (2003). Aberrant expression of peroxiredoxin subtypes in neurodegenerative disorders. Brain Res..

[39] Szeliga M. (2022). Comprehensive analysis of the expression levels and prognostic values of PRDX family genes in glioma. Neurochem. Int..

[40] Yun H.M., Park K.R., Kim E.C., Hong J.T. (2015). PRDX6 controls multiple sclerosis by suppressing inflammation and blood brain barrier disruption. Oncotarget.

[41] Garcia-Bonilla L., Iadecola C. (2012). Peroxiredoxin sets the brain on fire after stroke. Nat. Med..

[42] Shichita T., Hasegawa E., Kimura A., Morita R., Sakaguchi R., Takada I., Sekiya T., Ooboshi H., Kitazono T., Yanagawa T. (2012). Peroxiredoxin family proteins are key initiators of post-ischemic inflammation in the brain. Nat. Med..

[43] Shanshan Y., Beibei J., Li T., Minna G., Shipeng L., Li P., Yong Z. (2017). Phospholipase A2 of Peroxiredoxin 6 Plays a Critical Role in Cerebral Ischemia/Reperfusion Inflammatory Injury. Front. Cell Neurosci..

[44] Kraig R.P., Chesler M. (1990). Astrocytic acidosis in hyperglycemic and complete ischemia. J. Cereb. Blood Flow Metab..

[45] Vázquez-Medina J.P., Tao J.Q., Patel P., Bannitz-Fernandes R., Dodia C., Sorokina E.M., Feinstein S.I., Chatterjee S., Fisher A.B. (2019). Genetic inactivation of the phospholipase A_2_ activity of peroxiredoxin 6 in mice protects against LPS-induced acute lung injury. Am. J. Physiol. Lung Cell Mol. Physiol..

[46] Ambruso D.R. (2013). Peroxiredoxin-6 and NADPH oxidase activity. Methods Enzymol..

[47] Gosbell A.D., Stefanovic N., Scurr L.L., Pete J., Kola I., Favilla I., de Haan J.B. (2006). Retinal light damage: Structural and functional effects of the antioxidant glutathione peroxidase-1. Investig. Ophthalmol. Vis. Sci..

[48] Wang G., Zhong W.C., Bi Y.H., Tao S.Y., Zhu H., Zhu H.X., Xu A.M. (2019). The Prognosis Of Peroxiredoxin Family In Breast Cancer. Cancer Manag. Res..

[49] Albright B., Dhaher R., Wang H., Harb R., Lee T.W., Zaveri H., Eid T. (2017). Progressive neuronal activation accompanies epileptogenesis caused by hippocampal glutamine synthetase inhibition. Exp. Neurol..

[50] Reinehr R., Görg B., Becker S., Qvartskhava N., Bidmon H.J., Selbach O., Haas H.L., Schliess F., Häussinger D. (2007). Hypoosmotic swelling and ammonia increase oxidative stress by NADPH oxidase in cultured astrocytes and vital brain slices. Glia.

[51] Görg B., Karababa A., Shafigullina A., Bidmon H.J., Häussinger D. (2015). Ammonia-induced senescence in cultured rat astrocytes and in human cerebral cortex in hepatic encephalopathy. Glia.

[52] Lu K., Zimmermann M., Görg B., Bidmon H.J., Biermann B., Klöcker N., Häussinger D., Reichert A.S. (2019). Hepatic encephalopathy is linked to alterations of autophagic flux in astrocytes. EBioMedicine.

[53] Kim J.E., Kim Y.J., Kim J.Y., Kang T.C. (2014). PARP1 activation/expression modulates regional-specific neuronal and glial responses to seizure in a hemodynamic-independent manner. Cell Death Dis..

[54] Kim J.E., Kwak S.E., Choi S.Y., Kang T.C. (2008). Region-specific alterations in astroglial TWIK-related acid-sensitive K+-1 channel immunoreactivity in the rat hippocampal complex following pilocarpine-induced status epilepticus. J. Comp. Neurol..

